# [Retracted] Expression of caspase‑3, Bax and Bcl‑2 in hippocampus of rats with diabetes and subarachnoid hemorrhage

**DOI:** 10.3892/etm.2024.12704

**Published:** 2024-09-02

**Authors:** Xin He, Jiankui Sun, Xiaoyu Huang

Exp Ther Med 15:873–877, 2018; DOI: 10.3892/etm.2017.5438

Following the publication of the above paper, a concerned reader drew the Editor's attention to the fact that unexpected areas of similarity / identity were noted in the TUNEL assay data shown in Fig. 1A-D on p. 874, comparing data both within certain panels and data between them, such that the matches identified in the question were difficult to attribute to coincidence.

Following an internal enquiry, the Editor of *Experimental and Therapeutic Medicine* has been able to verify the claims made by the concerned reader; therefore, in view of the abovementioned anomalies that were identified and owing to an overall lack of confidence in the presented data, the Editor has decided that the paper should be retracted from the Journal. The authors were asked for an explanation to account for these concerns, but the Editorial Office did not receive a reply. The Editor apologizes to the readership of the Journal for any inconvenience caused.

